# Aryl hydrocarbon receptor (AHR) is a novel druggable pathway controlling malignant progenitor proliferation in chronic myeloid leukemia (CML)

**DOI:** 10.1371/journal.pone.0200923

**Published:** 2018-08-09

**Authors:** Melanie Gentil, Patricia Hugues, Christophe Desterke, Gladys Telliam, Ivan Sloma, Lucas E. B. Souza, Seda Baykal, Jerome Artus, Frank Griscelli, Agnes Guerci, Hyacinthe Johnson-Ansah, Adlen Foudi, Annelise Bennaceur-Griscelli, Ali G. Turhan

**Affiliations:** 1 Inserm U935, Villejuif, France; 2 University Paris Sud, Faculty of Medicine, Le Kremlin Bicêtre, France; 3 Service d’Hématologie, Hôpital Bicêtre and Paul Brousse, Le Kremlin Bicêtre and Villejuif, France; 4 Dokuz Eylul University Medical School, Medical Biology and Genetics Dept, Izmir, Turkey; 5 CHU de Nancy, Nancy, France; 6 CHU Caen, Department of Hematology, Caen, France; 7 Institut Federatif d’Hématologie Paris Sud (IFHIPS), APHP and Service d’Hématologie Bicêtre and Paul Brousse, Villejuif, France; Universita degli Studi di Firenze, ITALY

## Abstract

Aryl Hydrocarbon Receptor (AHR) is an ubiquitous basic helix-loop-helix transcription factor, which is ligand-activated and involved in numerous biological processes including cell division, cell quiescence and inflammation. It has been shown that AHR is involved in normal hematopoietic progenitor proliferation in human cells. In addition, loss of AHR in knockout mice is accompanied by a myeloproliferative syndrome-like disease, suggesting a role of AHR in hematopoietic stem cell (HSC) maintenance. To study the potential role of AHR pathway in CML progenitors and stem cells, we have first evaluated the expression of AHR in UT-7 cell line expressing BCR-ABL. AHR expression was highly reduced in UT-7 cell expressing BCR-ABL as compared to controls. AHR transcript levels, quantified in primary peripheral blood CML cells at diagnosis (n = 31 patients) were found to be significantly reduced compared to healthy controls (n = 15). The use of StemRegenin (SR1), an AHR antagonist, induced a marked expansion of total leukemic cells and leukemic CD34+ cells by about 4- and 10-fold respectively. SR1-treated CML CD34+ cells generated more colony-forming cells and long-term culture initiating cell (LTC-IC)–derived progenitors as compared to non-SR1-treated counterparts. Conversely, treatment of CML CD34+ cells with FICZ, a natural agonist of AHR, induced a 3-fold decrease in the number of CD34+ cells in culture after 7 days. Moreover, a 4-day FICZ treatment was sufficient to significantly reduce the clonogenic potential of CML CD34+ cells and this effect was synergized by Imatinib and Dasatinib treatments. Similarly, a 3-day FICZ treatment contributed to hinder significantly the number of LTC-IC-derived progenitors without synergistic effect with Imatinib. The analysis of molecular circuitry of AHR signaling in CML showed a transcriptional signature in CML derived CD34+ CD38- primitive cells with either low or high levels of AHR, with an upregulation of myeloid genes involved in differentiation in the “AHR low” fraction and an upregulation of genes involved in stem cell maintenance in the “AHR high” fraction. In conclusion, these findings demonstrate for the first time that down-regulation of AHR expression, a major cell cycle regulator, is involved in the myeloproliferative phenotype associated with CML. AHR agonists inhibit clonogenic and LTC-IC-derived progenitor growth and they could be used in leukemic stem cell targeting in CML.

## Introduction

Chronic myeloid leukemia (CML) is a clonal malignancy of the hematopoietic stem cell, characterized by a massive expansion of hematopoietic progenitors and their differentiated progeny [1] [2]. During the last two decades, major progress has been obtained in the understanding of CML pathophysiology, with the demonstration of several signalling pathways involved such as STAT5, PI-3K/AKT, RAS. CML is also characterized by a major genomic instability with abnormal DNA repair due to alteration of DNA repair mechanisms [3] [4] [5].

The elucidation of these signaling abnormalities allowed identification of novel targets, especially in the context of targeting leukemic stem cells (LSC) (PML, ALOX5a, SMO, STAT5). Indeed, despite the major effect of the tyrosine kinase inhibitors (TKI) on the elimination of the bulk leukemic cells, these drugs appeared unable to eradicate LSC [6] [7] which persist [2] and lead to relapses upon TKI discontinuation [8]. In our studies aiming to identify novel signaling pathways involved by the generation of CML, we have identified AHR as a novel gene down regulated by BCR-ABL. We report here the implication of the AHR pathway in the behaviour of progenitor and stem cell compartment in primary CML samples.

## Materials and methods

### UT-7 and UT-7-BCR-ABL

UT-7 cell line as well as its BCR-ABL-expressing counterpart UT-7/11 were generated and cultured as previously described [9].

### Compounds

StemRegenin 1 (Cellagen Technology) was used at concentrations ranging from 0.01μM to 1 μM. FICZ (6-Formylindolo (3,2-b) carbazole) was used at concentrations ranging from 20 to 600 nM. Imatinib was used at 1μM and Dasatinib at 5nM.

### Primary CML samples

Bone marrow and peripheral blood mononuclear cells (PBMC) were obtained from patients with CML at diagnosis and from healthy donors with the informed consent and according to the Declaration of Helsinki. All samples, including those from patients and those from healthy donors, were fully de-identified prior to access by any of the authors. All samples, including those from patients and those from healthy donors, were collected as part of routine care.

31 patients were included in the study. In eight of these patients, blood samples obtained at major molecular response were also analyzed. Control samples included PBMC from 15 healthy donors. Mononuclear cells were isolated from blood by Ficoll gradient. CD34+ cells were purified from mononuclear cells by immunomagnetic column separation according to the manufacturer’s recommandations (Miltenyi Biotech).

### Flow cytometry analyses and cell sorting

CD34+ cells were purified from mononuclear cells by immunomagnetic column separation (Miltenyi Biotech) and were washed once in PBS and labeled with anti CD34-APC (Miltenyi Biotech) and CD38-PE (Miltenyl Biotech). Dead cells were excluded using DAPI stain (1 μg/mL). Monoclonal isotype–matched control antibodies were used to determine the level of non-specific signal. CD34+CD38- cells were sorted using a FacsAria (BD-Biosciences) flow cytometer [10].

### Serum-free culture

CD34+ cells were cultured in Iscove modified Dulbecco medium (IMDM, Gibco) supplemented with a serum substitute containing bovine serum albumin, insulin and transferrin (BIT) (Stemcell Technologies) and 10^−4^ M 2-mercaptoethanol (Sigma, St Louis, MO, USA), supplemented with recombinant human Flt3-ligand (100 ng/mL) Stem Cell Factor (100 ng/ml) recombinant human interleukin-3 (20 ng/mL) recombinant human interleukin-6 (10ng/mL) and granulocyte-colony-stimulating factor (10 ng/mL). All growth factors were purchased from Stem Cell Technologies.

### Colony forming cell (CFC) and long-term culture initiating cell (LTC-IC) assays

Human CD34+ cells were plated at a concentration of 500 cells/mL in semi-solid methylcellulose cultures for colony-forming cell (CFC) assay (H4435 Stem Cell Technologies). The type and number of CFC progenitors were scored 7 and 14 days later using an inverted microscope. Similarly, CML-derived CD34+ cells were subjected to 100 μM SR1 for 4, 7 and 14 days and assayed for CFC content in similar conditions as above. CML CD34+ cells subjected to SR1 treatment for 4 or 7 days were assayed for LTC-IC content as previously described [6]. Briefly, CD34+ were plated with a monolayer of MS5 stromal cells and cultured in long-term initiating medium (H5100 Stem cell Technologies) during 5 weeks in 96-well plates. After 5 weeks, all wells were sacrificed by trypsinization, washed and plated in methylcellulose cultures for colony-forming cell (CFC) assay and quantification of the number of LTC-IC as described previously [6].

### Quantitative real time reverse transcription and polymerase chain reaction

RNA from peripheral blood mononuclear cell stored in Trizol (Invitrogen) was extracted on chloroform and RNA from 34+ cells and 34+38- cells was extracted on Picopure Kit (Life Technologies, Carlsbad, CA, USA). One microgram RNA was used per reverse transcription reaction using the Superscript2 (Invitrogen). Q-PCR measurements of transcripts were taken using TaqMan® Universal PCR Master Mix (ThermoFisher). Each sample was tested twice for the expression of the transcripts.

AHR expression was analyzed by real time q-RT-PCR using Applied Biosystems® 7500 Real-Time PCR Systems (ThermoFisher) using primers sets purchased from Applied Biosystems.

BCR-ABL and ABL transcripts in cDNA were measured by Q-RT-PCR using (Taqman; AppliedBiosystems, Foster City, CA) according to standard procedures as previously described [6]. Briefly, a standard curve was generated for each assay using serial dilutions of linearized plasmid containing a BCR-ABL insert. Hence, the same plasmid was used for quantification of BCR-ABL and ABL, which should control for any variation in plasmid quantitation efficiency between BCR-ABL and ABL.

### Transcriptome datasets

Affymetrix Human Genome U133A 2.0 Array data matrix normalized by MAS5 software from GSE11889 [11] was downloaded on Gene Expression Omnibus website: (http://www.ncbi.nlm.nih.gov/geo/query/acc.cgi?acc=GSE11889), and annotated with annotation plateform GPL571 (http://www.ncbi.nlm.nih.gov/geo/query/acc.cgi?acc=GPL571). In this dataset samples belonging to the following experimental groups were analyzed: CD34^+^CD38^low^ hematopoietic progenitors from Healthy donors (GSM300146, GSM300132, GSM300136, GSM300140, GSM300128); CD34^+^CD38^low^ from patients with chronic myeloid leukemia (GSM300124, GSM300111, GSM300120, GSM300106, GSM300093, GSM300102, GSM300116).

### Bioinformatics microarray data analysis

AHR gene expression ranking in CD34+CD38low samples from dataset GSE11889 identified two groups of hematopoietic progenitors in CML pathology. Boxplot, two-sided Student *t* test with Welch correction were performed in R Software version 3.2.3. Predictive expression profile was performed with PAMr algorithm by supervision between the two groups of CML hematopoietic progenitors AHR related [12]. Heatplot was performed with made4 package [13] used correlation classification metric with average distance on differential expressed genes identified with PAMr algorithm. Expression profile containing predictive genes for CML patients which over-expressed AHR in their hematopoietic progenitors was used to realize a functional enrichment with Go-Elite Standalone software version 1.2 on the Gene Ontology Biological Process database included in Homo sapiens EnsMart77Plus (Ensembl–Biomart) update [14]. Functional interaction network was built with functional relations identified during enrichment analysis with Cytoscape software version 3.2.1 [15]: color of nodes in the network is relative to the Z-scores obtained during functional enrichment (starting from yellow for low Z-scores to purple for high Z-scores), sizes of the nodes are relative to the number of direct connections mapped on the network. Gene set enrichment analysis was performed between the 2 CML and AHR-dependent subgroups: standalone software version 2.2.2.0 was used with implementation of MSigDB 5.2 database [16].

### Statistical analyses

Results are shown as the arithmetic mean ± SD as indicated. Differences between groups were assessed using the Student *t* test or the Mann-Whitney test (*p* < 0.05 was considered significant).

## Results

### UT-7 cells expressing BCR-ABL exhibit reduced levels of AHR

**Fig 1:**
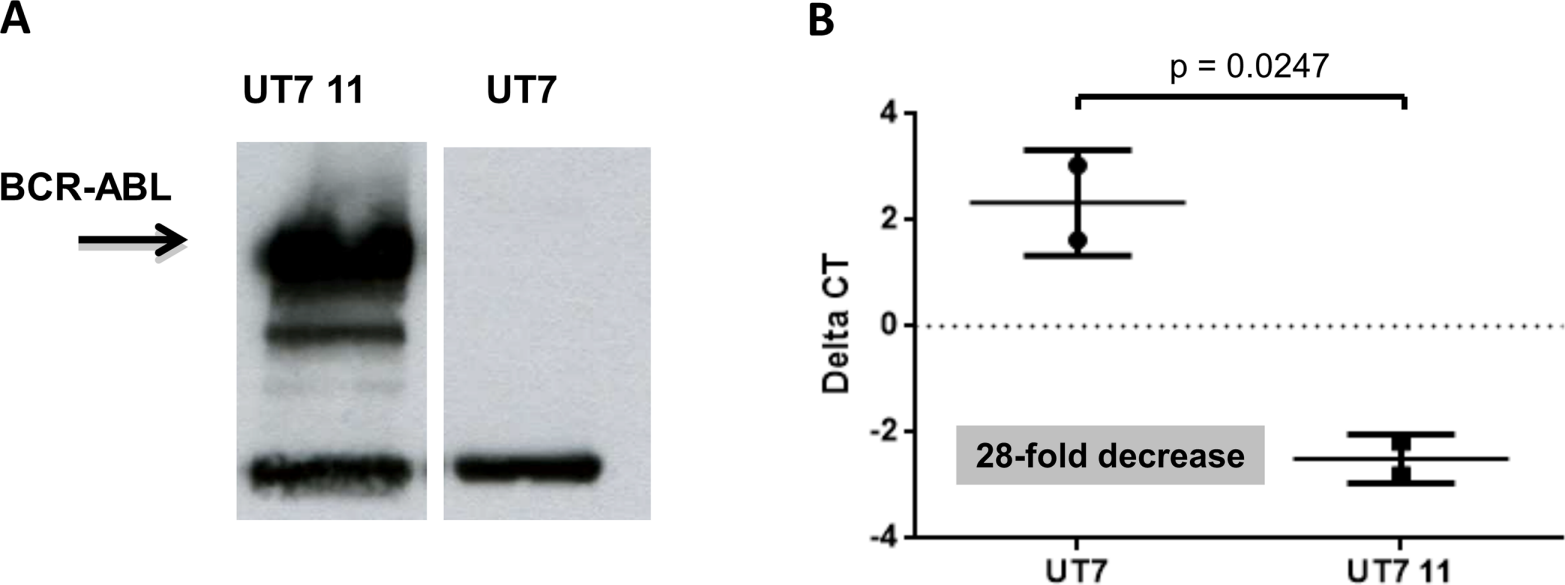
Expression of AHR in UT-7 cells and UT-7/11 cells. (A) Evaluation of the expression of BCR-ABL in the UT-7/11 cells as compared to the parental UT-7 cell line using Western blot analysis. As can be seen in this panel, BCR-ABL protein is highly expressed in the UT-7/11 cell line, with presence of some degradation products, which are visible. (B). Evaluation of the expression of the expression of AHR mRNA using Q-RT-PCR analyses. The expression of AHR is strongly downregulated in the BCR-ABL-expressing UT-7/11 cell line as compared to parental UT-7 cells. This difference is highly significant (t-test, p = 0.0247).

To determine the expression of AHR in BCR-ABL-expressing UT-7 cells we performed a quantitative RT-PCR assay. As shown in Fig 1A, UT-7/11 cell line exhibited high levels of BCR-ABL proteins as demonstrated by Western blot analyses. The analysis of AHR expression in the UT-7/11 cell line expressing high levels of BCR-ABL, showed reduced levels of AHR mRNA (28-fold decrease) as compared to parental UT-7 cells and this was highly significant (p = 0.0247, Student’s t-test, Fig 1B). These results prompted us to check its expression in primary CML cells.

### AHR mRNA expression is reduced in primary CML cells

**Fig 2:**
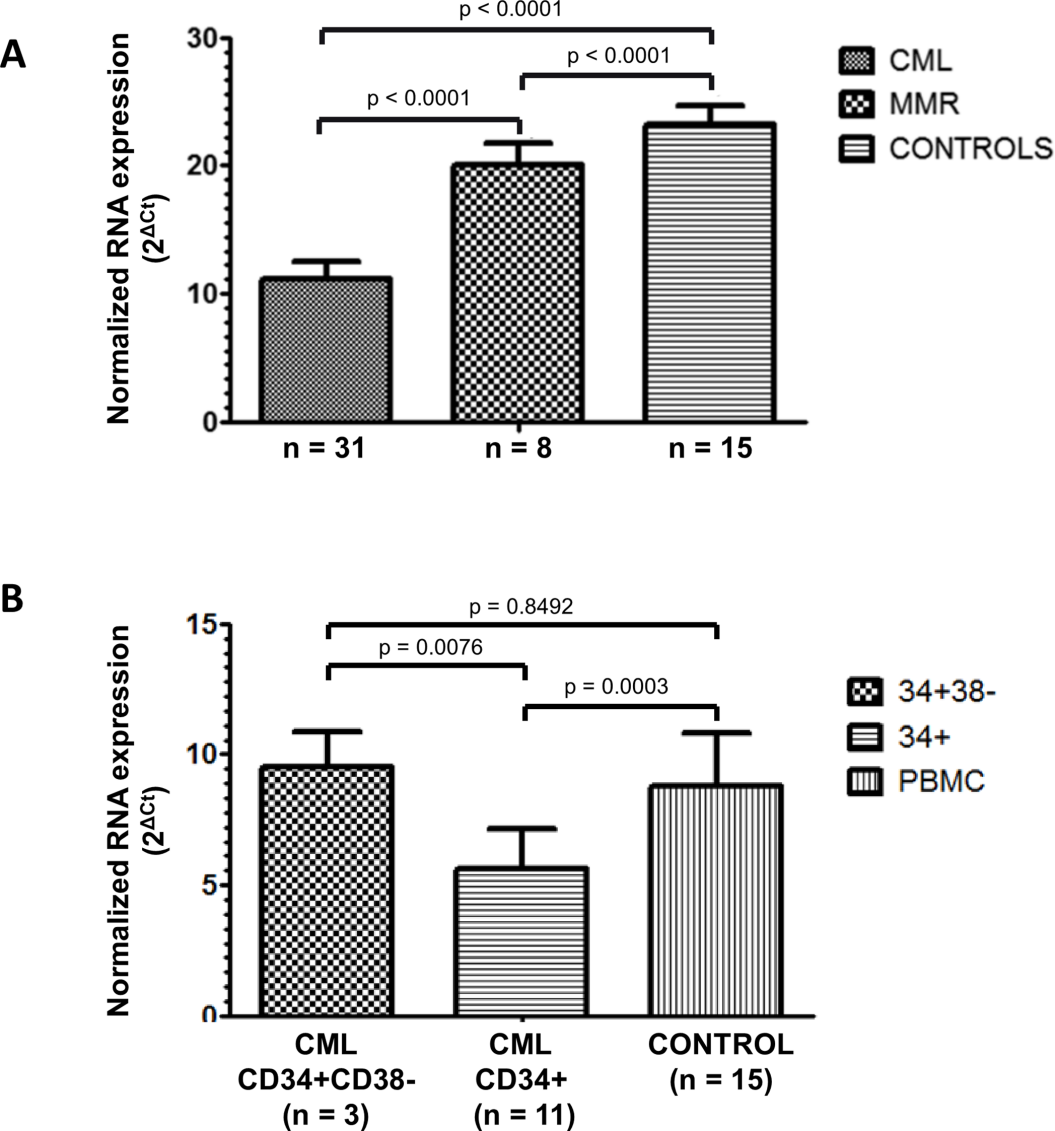
Expression of AHR in primary leukemic samples. (A) AHR transcript was quantified in primary peripheral blood CML cells at diagnosis (n = 31 patients) and were found to be very significantly reduced as compared to normal controls (n = 15) (p < 0.0001, ANOVA and Tukey’s post-hoc test). In 8 of these patients, AHR mRNA was quantified during TKI-induced major molecular response (MMR) and were found to increase towards levels found in controls in a statistically significant manner (p < 0.0001). Reported p values were adjusted for multiple comparisons. (B) AHR expression in CD34+ cells (n = 11) as well as in highly purified CD34+ CD38- cell populations (n = 3). Interestingly, AHR expression was reduced in leukemic CD34+ progenitors (n = 11) but not in more primitive leukemic CD34+ CD38- cells (n = 3, p = 0.0076) as compared to normal controls (n = 15).

For this analysis, we used leukemic blood samples of 31 patients with CML at diagnosis. As shown in Fig 2A, there was a significant reduction of AHR mRNA expression in CML patients at diagnosis as compared to healthy controls (n = 15) and this difference was highly significant (Fig 2A). Eight out of these 31 patients have been analyzed also after induction of a major molecular remission (MMR) obtained with TKI therapy. As can be seen in Fig 2A, in all of them, the level of AHR mRNA expression was significantly increased as compared to diagnosis, close to AHR levels observed in healthy controls and this difference was also highly significant (p < 0.0001, Fig 2A).

We have then asked whether there would be a differential expression of AHR in the more primitive hematopoietic stem cells as compared to bulk CD34+ population in CML patuents. In 3 patients, CD34+ CD38- cells were cell-sorted and analyzed for AHR expression. As can be seen in Fig 2B, the expression of AHR in the most primitive CML cells was found to be close to that seen in controls, whereas leukemic CD34+ cells exhibited low levels of AHR, with persistent statistically significant difference as compared to controls (p = 0.0003, Fig 2B).

### The AHR antagonist SR-1 induces a major expansion of leukemic CML cells in short term culture

**Fig 3:**
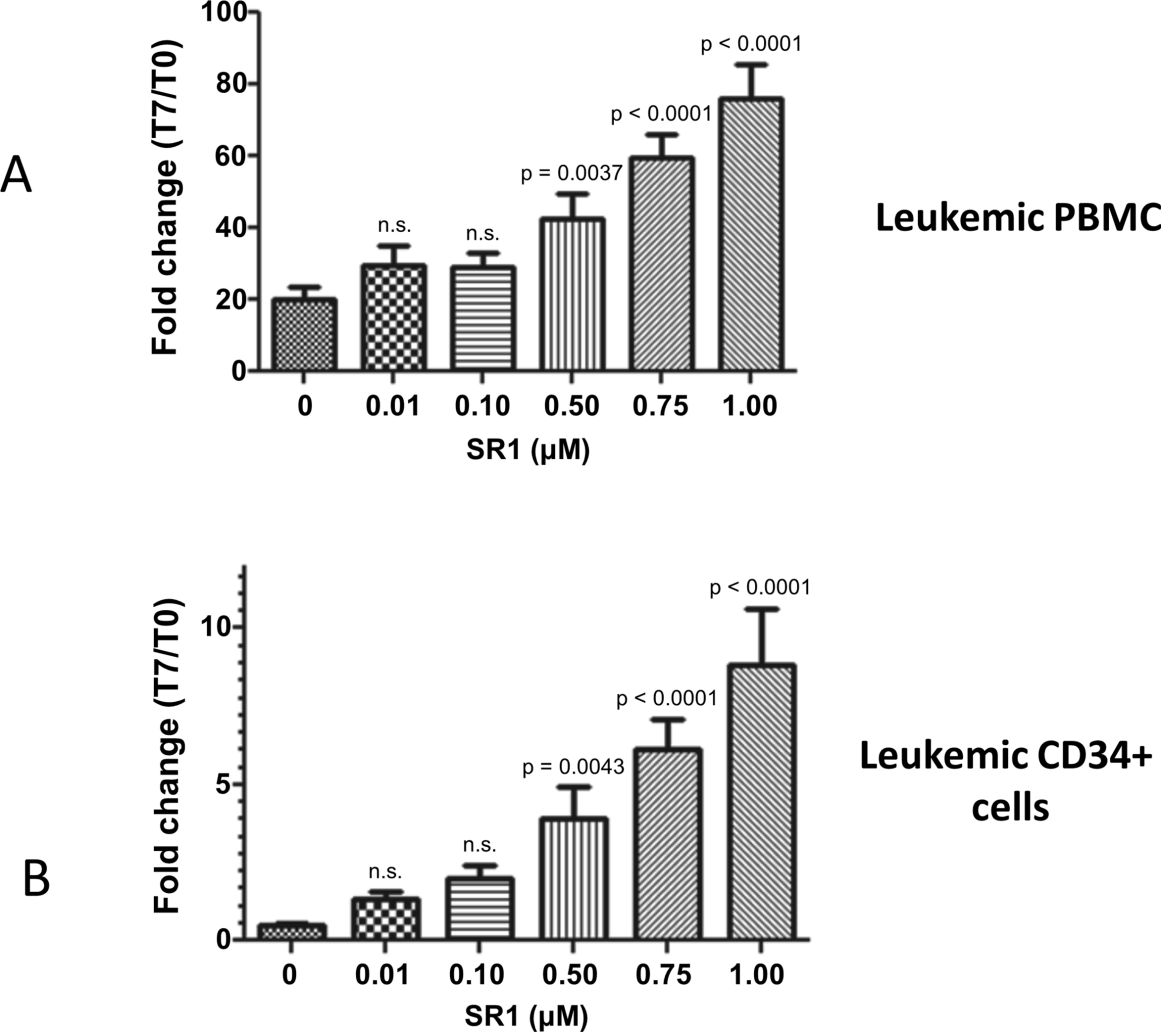
Analysis of the effects of AHR antagonist SR-1 on leukemic cell growth. A-In 7-day liquid cultures, the use of SR-1 at 0.75 μM induced a major expansion of leukemic cells by 60-fold as compared to conditions without SR-1 (p < 0.0001). B-SR-1 allowed expansion of leukemic CD34+ cells by 6-fold in 7 days, as compared to controls (p < 0.0001) n = 3 experiments, Mean + SD, Statistical test: ANOVA + Dunnett’s post-hoc test. Reported p values are related to comparison with the 0 uM dose. All p-values are adjusted for multiple comparisons. PBMC: Peripheral blood mononuclear cells.

As AHR is involved in maintaining cell quiescence in hematopoietic cells and given its reduced expression in CML cells, we asked whether a further inhibition of AHR could increase the myeloproliferative phenotype of CML cells at the level of progenitors and stem cells. For this purpose, we cultured CD34+ cells from CML patients in the presence of hematopoietic growth factors and StemRegenin (hereafter named SR1), a well-known AHR antagonist. We performed dose-response experiments to determine the optimal dose to be used on leukemic cells. As can be seen in Fig 3, when CML cells obtained at diagnosis either as non-purified PBMC (Fig 3A) or purified CD34+ cells (Fig 3B) there was a massive expansion of leukemic cells after 7 days of culture in the presence of SR1 (Fig 3A). This expansion was observed both in total leukemic PBMC (about 4-fold as compared to the numbers obtained at day 7 with the use of growth factors alone) and in leukemic CD34+ cells (about 10-fold as compared to the conditions with the use growth factors alone) in the presence of 1 μM SR1. We then asked whether this expansion at the level of differentiated leukemic cells would result in an expansion of leukemic progenitors and more importantly, of hematopoietic stem cells.

### Massive expansion of leukemic cells by SR-1 translates into expansion of leukemic progenitors and stem cells

**Fig 4:**
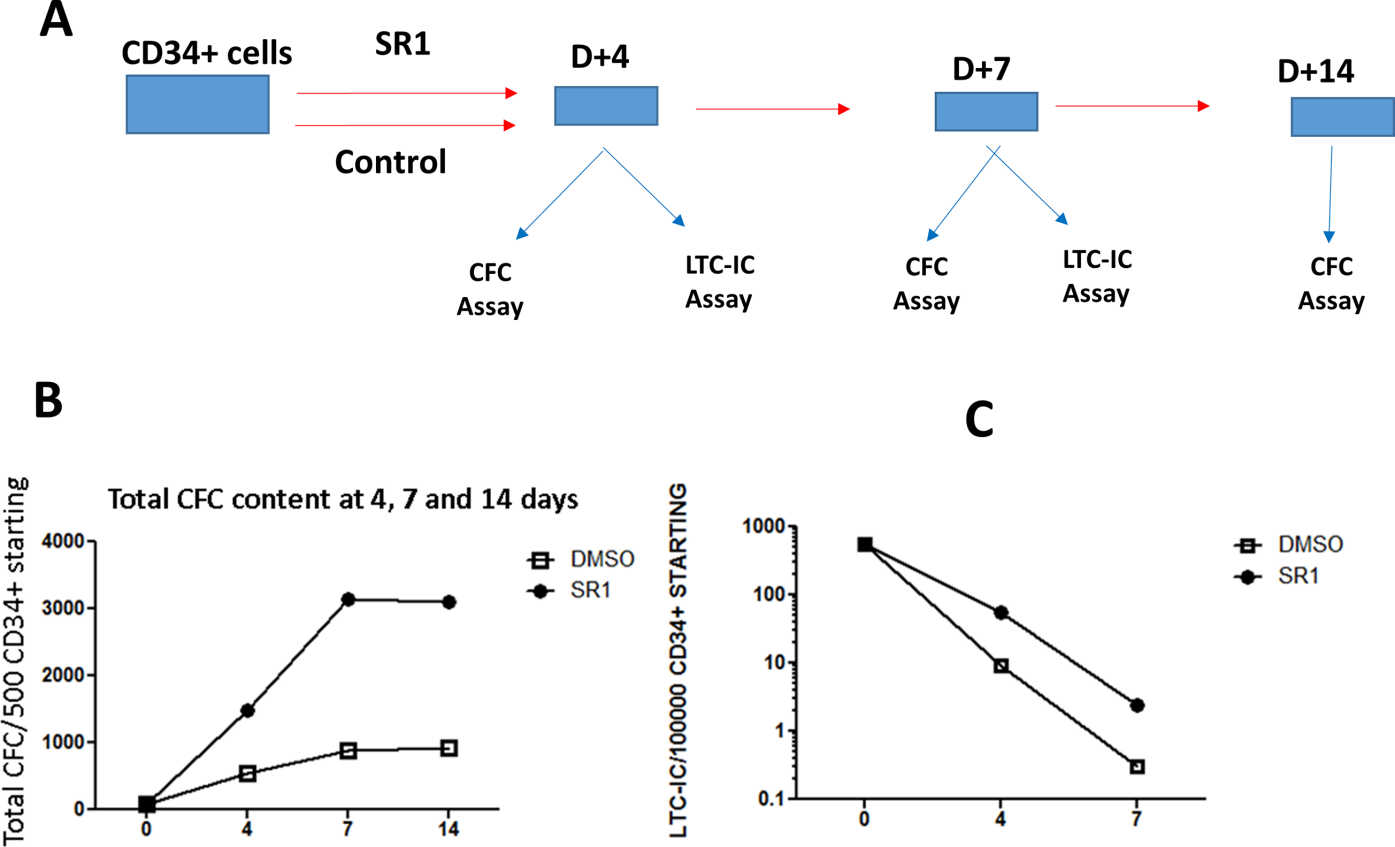
Analysis of the effects of AHR antagonist SR-1 on progenitor and leukemic stem cell growth. A-Experimental strategy used to evaluate the role of AHR signaling in expansion of clonogenic cells and LTC-IC-derived progenitors. B-CML CD34+ cells cultured with SR-1 for 7 and 14 days exhibited a massive expansion of leukemic CFC (> 3 fold increase), as shown in this representative experiment of 3. C -CD34+ cells expanded with SR-1 for 4 and 7 days followed by long-term culture initiating cells (LTC-IC) assays, revealed a massive expansion (> 10-Fold) of LTC-IC-derived progenitors. n = 3 experiments, a representative experiment is shown in the Fig.

To determine the effect of SR1-induced hematopoietic cell expansion at the level of CML progenitors and stem cells, we cultured CML-derived CD34+ cells in the presence of 1 μM SR1 or control DMSO for 4, 7 or 14 days and subjected treated cells to clonogenic assays (CFC and LTC-IC) (Fig 4A). As can be seen in Fig 4B, after 7 or 14 days of culture, total CFC content of SR1-expanded cells was increased by 3-fold as compared to CD34+ cells cultured in growth factors alone and DMSO (Fig 4B). This difference was noticeable starting at 4 days of culture and persisted for up to 14 days. This SR1-mediated expansion did not alter colony output as similar numbers of CFU-GM and BFU-E colonies were observed with and without SR1 (data not shown).

To determine whether SR1-mediated AHR inhibition would also expand more primitive stem cells, we collected CD34+ cells after 4 and 7 days of culture with SR1 and performed LTC-IC assays as previously described [6]. As shown in Fig 4C, AHR inhibition via SR1 treatment in CD34+ cells led to a marked increase in the number of LTC-IC-derived progenitors both at day 4 and 7 by about 10- and 6-fold respectively (Fig 4C). These results demonstrate that AHR pathway restricts the proliferation of both leukemic progenitors and more primitive stem cell populations in CML.

### Effects of FICZ on the growth of leukemic progenitors and stem cells

**Fig 5:**
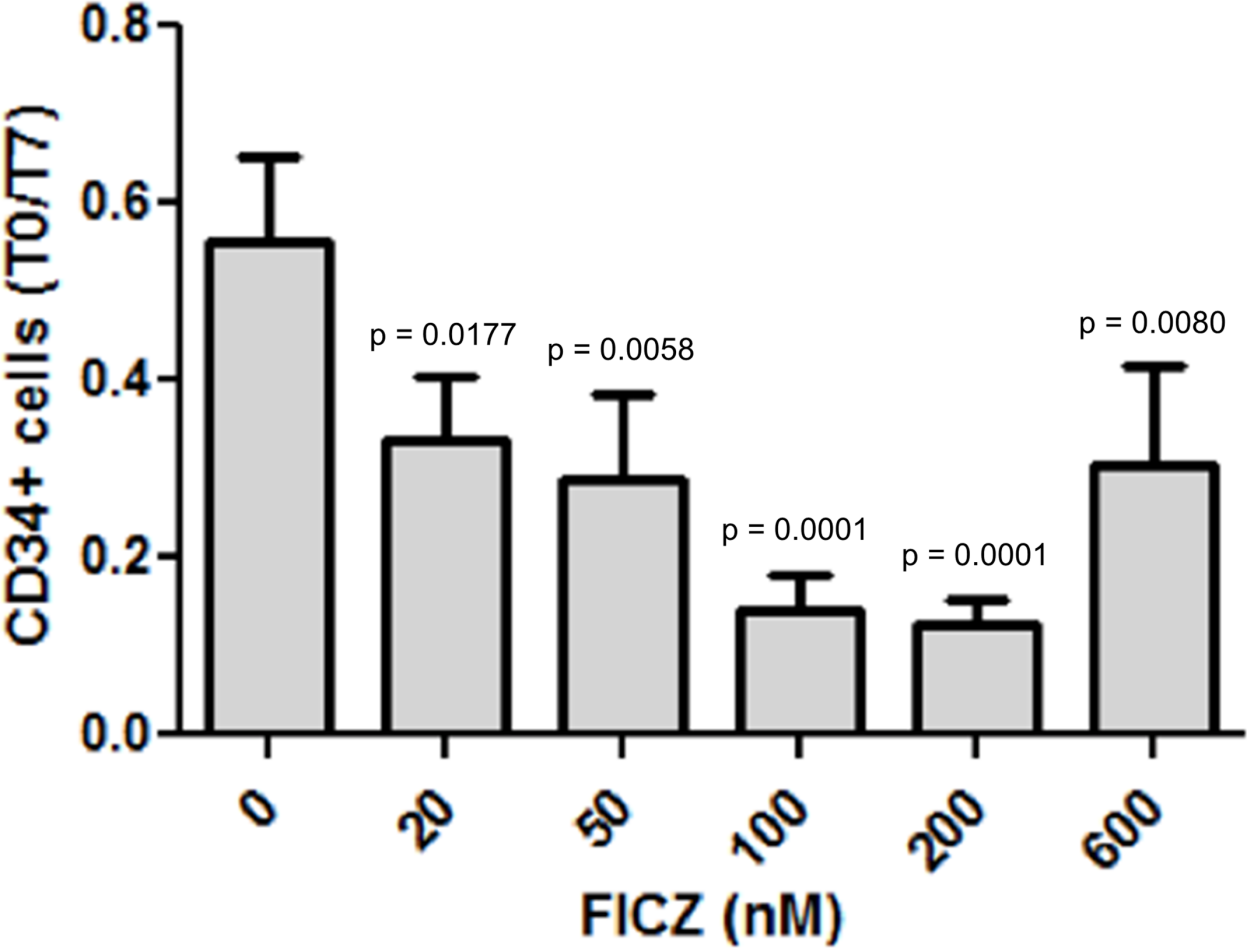
Evaluation of the role of AHR agonist FICZ in the growth of leukemic CD34+ cells. CML CD34+ cells were cultured in the presence of indicated concentrations of FICZ and the cell numbers at day7 were compared to day 0. The use of FICZ induced a decrease of the growth of CD34+ cells at day+7 with the most inhibitory effects observed at concentrations of 100–200 nm (p = 0.0001, n = 3 experiments, ANOVA + Dunnett’s post-hoc test). Reported p-values are related to comparisons performed with the 0 nM dose. All p-values are adjusted for multiple comparisons.

**Fig 6:**
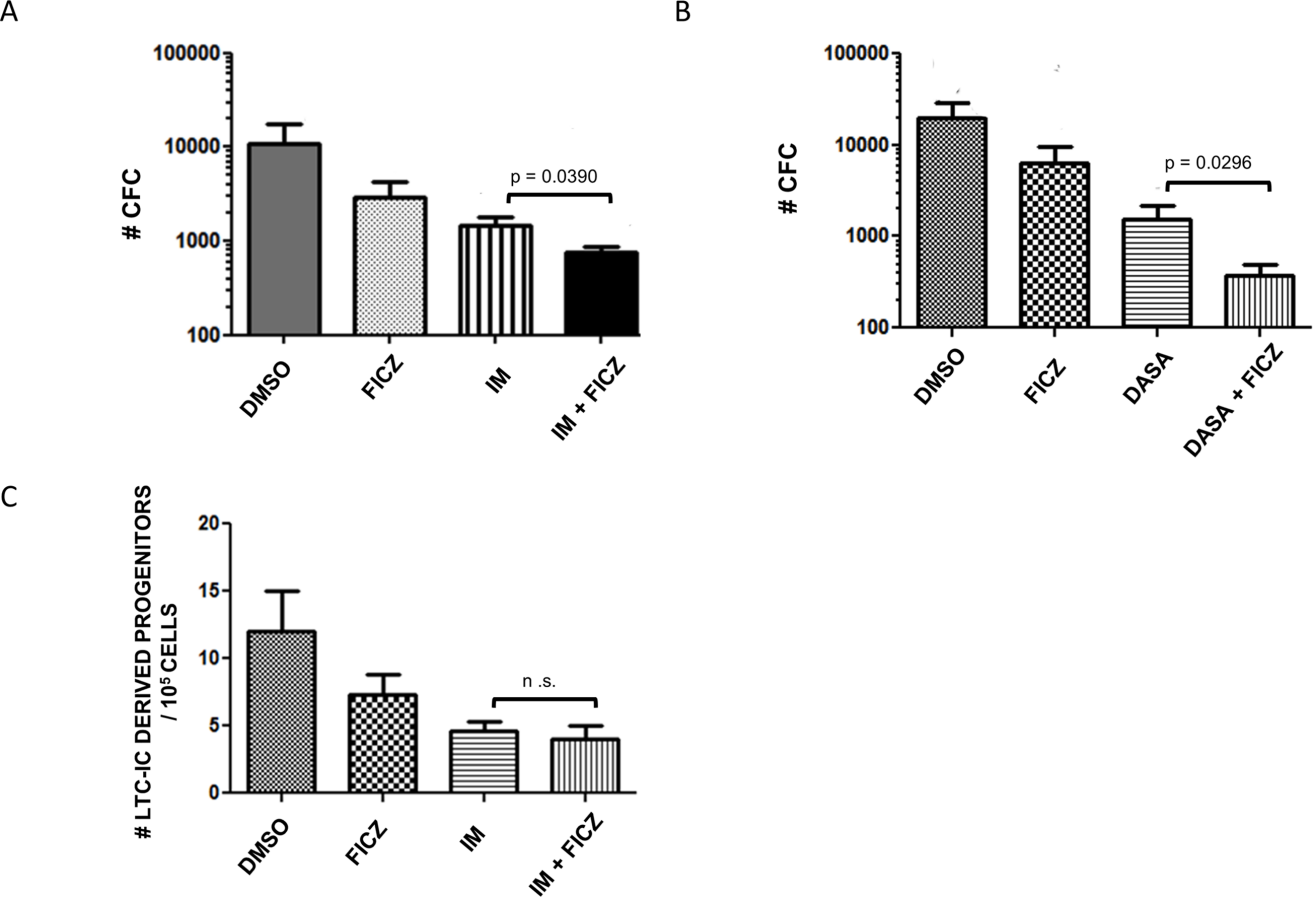
Evaluation of the clonogenic potential of day-4 FICZ-cultured CML CD34+ cells. A significant reduction of leukemic clonogenic potential was observed with a synergistic profile of FICZ with Imatinib (p = 0.0309, Panel A) and Dasatinib (p = 0.0292. (Panel B). Similarly, in three patients, LTC-IC assays started using 3-day FICZ-treated cells showed a significant growth inhibition of LTC-IC-derived clonogenic progenitors without synergistic profile with Imatinib. (Panel C). n = 3 experiments. CFC: Colony forming cells; IM: Imatinib; DASA: Dasatinib; DMSO: Dimethysulfoxide condition in which cells were cultured in the presence of DMSO at 0.01%.

Given the fact that AHR inhibition enhanced progenitor and stem cell proliferation in CML patients, its activation could be a potential therapeutic target to reduce and eliminate leukemic stem cells. To check this hypothesis, we used the 6-Formylindolo (3,2-b) carbazole (FICZ), a natural agonist of AHR, to determine its effects on leukemic progenitor and stem cell proliferation. As expected, CML CD34+ cells cultured in the presence of 100 nM FICZ proliferated significantly less than control cultures in the presence of DMSO (0.01%) as noted by the 3-fold decrease of the number of CD34+ cells at day 7 (Fig 5). We then analyzed the inhibitory effect of FICZ at the level of clonogenic progenitors and LTC-IC-derived progenitors. As can be seen in Fig 6, a 4-day culture of CML CD34+ cells in the presence of FICZ was sufficient to significantly reduce their clonogenic potential in synergy with Imatinib (p = 0.039, Fig 6A) and Dasatinib (p = 0.0296, Fig 6B). A 3-day FICZ treatment contributed to reduce significantly the number of LTC-IC-derived progenitors but this effect was not found to be synergistic with Imatinib (Fig 6C).

#### Deregulation of AHR expression in CML CD34^+^CD38^low^ compartment and its functional consequences

**Fig 7:**
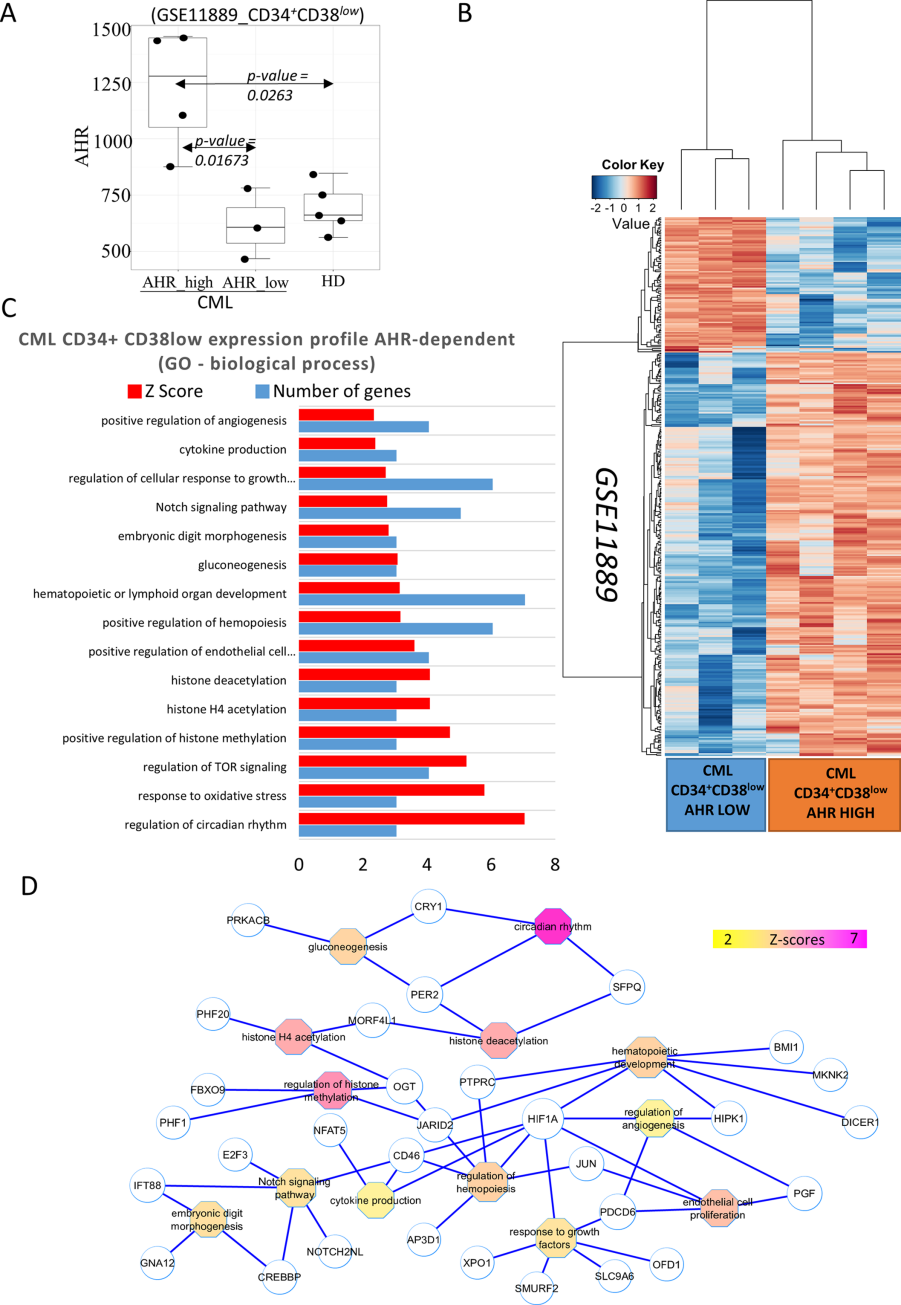
Transcriptome analysis of primitive CML CD34^+^CD38^low^ hematopoietic cells expressing low or high AHR expression. A: Boxplot of AHR gene expression in CD34^+^CD38^low^ cells from CML patients (2 subgroups of patients: AHR-low and AHR-high) in comparison of CD34^+^CD38^low^ cells from healthy donors (HD), p-values were calculated by comparing sample groups two by two with 2 sided Student t test and Welch correction; B: Heatmap of the predictive gene expression profile between the 2 CML patients subgroups (AHR-dependent: AHR-low and AHR-high) in CD34^+^CD38^low^ hematopoietic compartment (352 genes) (unsupervised classification was performed with Pearson distances and average linkage); C: Functional enrichment histogram of predictive expression profile correlated to AHR expression in CD34^+^CD38^low^ cells from CML patients; Biological Process from Gene Ontology database was used to perform the enrichment (red bars represent Z-scores of the enriched functions; blue bars represent number of genes implicated in enriched function); D: functional interaction network of the AHR-related expression profile in CML hematopoietic progenitors (CD34^+^CD38^low^): functional relation connecting gene to function were extracted from Biological Process Gene Ontology Database (circle nodes: enriched genes; octagon nodes: enriched functions; color scale from yellow to purple: Z-scores of the functional enrichment).

In order to determine the potential role of the AHR signaling pathway in CML stem cells, we used a bioinformatics analysis of the published transcriptome data in CD34^+^CD38^low^ CML stem cells (dataset GSE11889). Expression of AHR specific Affymetrix probe ranked between samples of CD34^+^CD38^low^ hematopoietic progenitors allowed to distinguish two AHR-related subgroups of patients with chronic myeloid leukemia: one subgroup of patients with AHR expression comparable to expression in healthy donor samples (n = 3 of 7, subgroup CML AHR-low), and one subgroup of patients with an overexpression of AHR (CML AHR-high) in hematopoietic progenitors (n = 4 of 7) in comparison to healthy donor samples (two-sided Student test with Welch correction: p-value = 0.0263, Fig 7A). A significant differential expression was also found for AHR between the 2 subgroups of CML hematopoietic progenitors: CML AHR-high *versus* CML AHR-low (two-sided Student test with Welch correction: p-value = 0.01673, Fig 7A). Predictive Analysis of Microarray (PAMr) algorithm supervised for CML hematopoietic progenitors subgroups (CML AHR-high and CML AHR-low) allowed to identify a predictive expression profile of 352 genes (S1 Table). This gene profile was validated by unsupervised classification using parameters: Pearson distances and average linkage (Fig 7B). Information from Biological Process of Gene Ontology Database was used to perform functional enrichment of gene profile predicting CML hematopoietic progenitors with high expression of AHR (CML AHR-high). In this fraction, an important deregulation of functions related to stem cell biology and to hematopoietic development were found to be enriched, implicating genes involved in glucose metabolism, in epigenetic deregulation with especially JARID2 which is known to interact with PRC2 Polycomb complex. Amongst other pathways, circadian rhythm genes PER2 and CRY1 as well as NOTCH signaling pathway were involved (Fig 7C and 7D).

## Discussion

Several signaling pathways have been shown to be involved in the quiescence and proliferation of CML stem cells such as PML, Alox5a and STAT5 [17] [18] [19]. AHR is a transcription factor activated by several ligands including toxins such as dioxins leading to the cell cycle arrest of target cells [20]. More recently, the major role of this pathway in hematopoiesis, carcinogenesis and tumor immunity has been discovered [21] [22] [23]. AHR has also been described as a major cell cycle regulator controlling quiescence status of HSC as its antagonists such as StemRegenin (SR1) allow ex-vivo expansion of hematopoietic progenitors [24]. The role of AHR in CML pathophysiology has not been studies so far. We report here for the first time the down-regulation of the expression of AHR in primary leukemic cells in CML. These results have been shown in a large number of patients with CML at diagnosis, suggesting a major participation of this down-regulation to the myeloproliferative phenotype as this has been shown in AHR-/- mice [25] [26]. These findings had two major implications, the first being the potential of the use of this pathway for potentiation of the myeloproliferative phenotype observed naturally in CML progenitors. This was achieved by the use of SR1, a well-known AHR antagonist the use of which has been shown to lead to a major amplification of normal progenitors and stem cells [24] The use of SR1 in liquid cultures including either total leukemic cells or purified CD34+ cells led to a major amplification of these cells by x 4-fold and by x10-fold respectively (Fig 4). These results are important as they represent the first pharmacological tool to amplify BCR-ABL-expressing primary leukemic cells in patients with CML, a goal which has been achieved previously only by the use of gene transfer techniques using NUP98-HOXA10 gene fusion transduced to CML progenitors [27]. The second implication was to determine if the major expansion of differentiated CML cells by SR1 would result in a significant expansion of more primitive compartments such as CML progenitors and stem cells. To this purpose, we have cultured primary CD34+ CML cells for 4, 7 and 14 days and tested their clonogenic and LTC-IC potential as compared to CD34+ cells cultured without SR1. As can be seen in Fig 4, after 4 days of culture in the presence of SR1, there was a major increase of CML progenitors recovered from these cultures (x3 fold) This effect was further increased when the cells cultured for 7 days and 14 days (x10 fold) suggesting a cumulative effect of AHR inhibition during this period. We then performed from the two time points of cultures with SR1, at day+4 and day+7 a long-term culture initiating cell assay to determine if the cells that we have cultured with SR1 lost their stem cell potential. As can be seen in Fig 4B, there was a major increase of LTC-IC-derived progenitors in cultures initiated with SR1-expanded cells, for either 4 or 7 days as compared to controls. These results have clearly established that AHR antagonism is a powerful way of amplifying CML stem cells.

AHR signaling can also be targeted by the use of AHR agonists. We show here that the AHR agonist FICZ inhibits the proliferation of leukemic CD34+ cells (Fig 5). This inhibitory effect can also be seen at the level of leukemic progenitors and pore primitive, LTC-IC-derived progenitors (Fig 6), suggesting that clinical grade AHR agonists could be of future use in targeting CML stem cells [6]. Interestingly, with higher doses of AHR, we have found a less inhibitory effect on the growth of CD34+ cells, suggesting that at higher doses FICZ could have a stimulatory effect on cell cycle depending on the amount of AHR expressed in the leukemic cells.

The role of AHR pathway in normal hematopoietic progenitors and stem cells has been studied mainly in mice [25] [26] and more recently in human hematopoiesis [24]. It has been shown that AHR -/- mice develop a myeloproliferative syndrome-like disorder, suggesting that AHR pathway plays a major role in controlling progenitor proliferation which is a major characteristic of CML. In order to study the potential implication of the AHR signaling the more primitive CML stem cells; we have performed a bioinformatics analysis of the gene profiling data of CML CD34+CD38-cells. This analysis shows that AHR controls fundamental aspects of stem cell biology including hypoxia-associated signaling, circadian rhythm genes and Notch signaling pathway in CML. This analysis also revealed that with regard to AHR expression there are two subgroups of CML stem cells, including a subgroup with high AHR and a subgroup with low AHR expression. The gene signature of these two subgroups appears different (S1 Table). Theoretically, CML stem cells with high AHR expression should activate potentially genes involved in quiescence. In fact, FGFR expression which is involved in the quiescence of hematopoietic stem cells [28] has been found to be upregulated in the “AHR high” fraction of leukemic stem cells (S1 Fig). Similarly, genes whose expression is involved in hypoxia “reactome” involved in stem cell maintenance, has been shown to be upregulated in “AHR high” population of CML stem cells.

On the other hand, the genes involved in myeloid differentiation and DNA replication (S1 Fig) were found to be upregulated in the “AHR low” fraction suggesting strongly that AHR is involved in the control of myeloid proliferation in CML.

Overall, data reported here, demonstrate, for the first time to our knowledge that AHR pathway plays a major role on the generation of the myeloproliferative phenotype associated with CML. We also show in this work that AHR pathway could be a very attractive target for eradication of CML progenitors and stem cells, in association with TKI. The effects that we have observed using AHR antagonism in CML progenitors and stem cells represent certainly the global results on the CD34+ cells expressing low versus high AHR expression. From this regard, it would be expected that high AHR expressing cells would undergo an entry into cell cycle leading to an expansion of progenitors and differentiated cells, with minimal effect on AHR low cells. On the other hand, the use of AHR agonists would have a preferential effect on “AHR low” cells, forcing them into a proliferative phenotype facilitating their elimination by TKI. Further experiments are required explore directly these hypotheses in CML.

## Supporting information

S1 Fig Gene set enrichment analysis performed on transcriptome of CD34+CD38^low^ cells from CML patients between the two subclasses with AHR dependency. A: Gene sets enriched in CML CD34+CD38^low^ hematopoietic cells according to low AHR expression;
B: Gene sets enriched in CD34+CD38l^ow^ cells of CML patients which harbored a high level expression of AHR as compared in CD34+CD38^low^ cells which harbored a low level expression of AHR (NES: normalized enrichment score).
(TIF)

S1 Table Predictive gene expression profile between two subclasses of CML CD34+CD38^low^ cell samples according to their AHR-low and AHR-high status. This Table describes in the first column: official gene symbols; in 2^nd^ column: ranking value of each gene as predictor to discriminate the 2 subclasses during learning machine process, 3^rd^ column describes calculated fold change between AHR-High subclass and AHR-Low subclasses, and last column, describe the description of the corresponding genes.
(XLSX)
