# Supplementary figures and images for: Aryl hydrocarbon receptor (AHR) is a novel druggable pathway controlling malignant progenitor proliferation in chronic myeloid leukemia (CML)
Source: PLoS One. 2018 Aug 9;13(8):e0200923. doi: 10.1371/journal.pone.0200923 (PMC6084853; doi:10.1371/journal.pone.0200923)

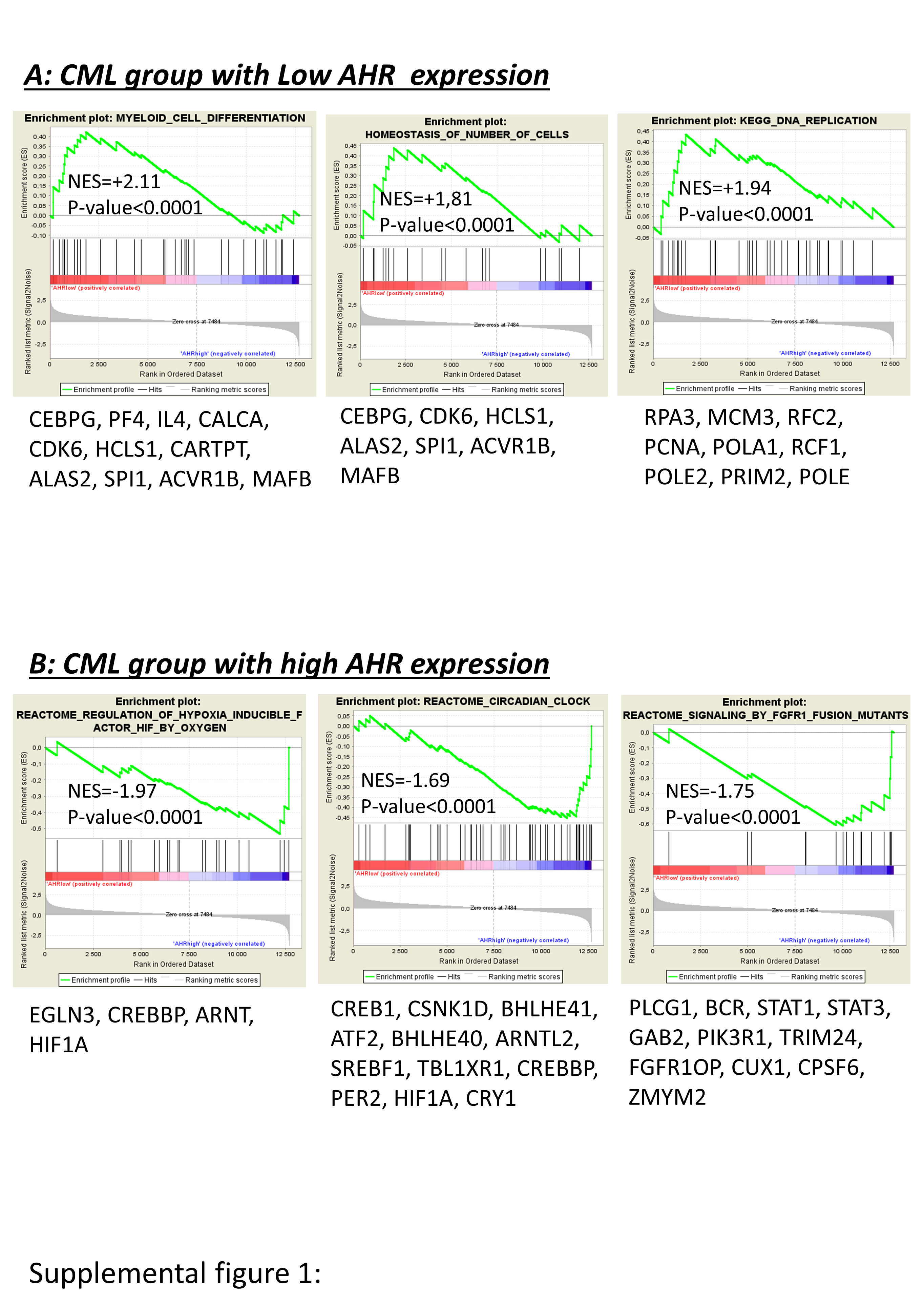

Supplement: S1 Fig — A: Gene sets enriched in CML CD34+CD38low hematopoietic cells according to low AHR expression; B: Gene sets enriched in CD34+CD38low cells of CML patients which harbored a high level expression of AHR as compared in CD34+CD38low cells which harbored a low level expression of AHR (NES: normalized enrichment score). (TIF) [file pone.0200923.s001.tif]
